# Disparities in three critical maternal health indicators amongst Muslims: Vis-a-vis the results reflected on National Health Mission

**DOI:** 10.1186/s12889-022-12662-7

**Published:** 2022-02-09

**Authors:** Md Illias Kanchan Sk, Balhasan Ali, Mohai Menul Biswas, Mrinal Kanti Saha

**Affiliations:** 1 International Institute for Population Sciences, Mumbai, India; 2 Tata Institute of Social Sciences, Mumbai, India

**Keywords:** Inequality, Muslim, Poor, Maternal care, India

## Abstract

**Background:**

The post national health mission era has been recognized for India’s accelerating improvement in maternal health care utilization. Concurrent investigations with the purview of examining inequalities in maternal care utilisation have rigorously examined across various socio-economic groups, focusing on Muslim women. The present study examined socio-economic differentials in maternal health care utilisation among Muslims and the delineated factors which are contributing for these inequalities.

**Methods:**

Study used the data from National Family Health Survey (NFHS) conducted in 2005-06 and 2015-16. the present study applied concentration index and Wagstaff-type decomposition analysis to measure and decompose the inequality in maternal health services.

**Results:**

This study found that utilisation of full antenatal care (full ANC), skilled attendants at birth (SBAs) and postnatal care was increased during 2005-06 to 2015-16. However, the least improvement was observed in full antenatal care whereas substantial improvement was achieved in utilising skilled attendants at birth. Further, the poor and non-poor gap in maternal health care utilisation mostly prevailed among the educated, urban resident, other backward castes among Muslims. The inequality has been declined largely in SBA utilisation compared to full ANC and PNC, especially in the southern India. Higher education, mass media exposure, higher birth order and urban residence contribute and explain most of these inequalities in maternal care among Muslim women

**Conclusions:**

Despite the fact that free and cash benefitted health programmes, wealth, mass media exposure and education etc welfare programs benefitted a large number of citizens, it also produced most of the inequalities among Muslims in India. The results focus on the significance of wealth, education, and mass media exposure in bridging the socioeconomic gap in maternal health care utilization among Muslims.

## Background

Because of its crucial importance in maintaining safe motherhood and the sustenance and nutrition of infants, as well as the overall well-being of families and communities, fair and equal requirement of maternal health care services such as antenatal care (ANC), skilled birth assistance (SBA), and post-natal care (PNC) is an essential portion of the complete healthcare system [1]. Maternal mortality is the prime cause of death among women of reproductive age, with a higher occurrence in low-resource situations.Over the last few decades, particularly since the 1990s (launching year of the millennium development goals (MDGs), the worldwide society has experienced significant efforts to turn the condition, as well as unprecedented reductions in maternal mortality [2]. Target 3.1 of the Sustainable Development Goals (SDGs) asks for the global maternal mortality ratio (MMR) to be reduced to less than 70 per 100,000 by 2030, with no nation having an MMR greater than 140 per 100,000 [3]. The global maternal mortality ratio (MMR) fell by 44% between 1990 and 2015, from 385 to 216 maternal deaths per 100,000 live births [3]. Notwithstanding this achievement, the globe fell well short of the Millennium Development Goals aim of reducing global MMR by 75% by 2015 [2, 3].

The South Asian countries have also met the most significant success in reducing maternal mortality. In South Asia, maternal mortalityhas been reduced to 190 per 100,000 live births in 2013 from 530 per 100,000 live births in 1990 [4].

However, post-MDGs estimation reveals that huge disparities exist in the access and utilisation of health care services among and within the countries. The world health community has now added some new domains of overall health to combat the global health problemin pursuit of achieving the sustainable development goals (SDGs). Hence, the sustainable development goals, which were launched in 2015, have continued to focus on maternal and child health in addition to focussing on overall health and well-being [5].

India has been putting enormous efforts in reducing maternal death through improved accessibility of public health facilities and services for the last three decades. According to the Registrar General of India report, the Maternal Mortality Ratio (MMR) has been reduced to only 113 maternal deaths per 100,000 live births in 2020 from 212 maternal deaths per 100,000 live births in 2007 [6]. Despite achieving such significant improvements, huge disparity still exists across the states. Since the beginning of the century, maternal deaths have been distributed unevenly across Indian states with more than 600 deaths in the east and north-central region and between 300-400 deaths in north-western and southern region [6, 7]. In 2005, Government of India introduced the momentous health programme, called the National Rural Health Mission (NRHM), to reduce maternal and child mortality by improving maternal health care services with a particular focus on the marginalized sections of the society. The goal of NRHM is to provide equitable, inexpensive, and high-quality health care to the rural population, particularly the disadvantaged groups. The mission is focused on setting up a completely operational, community-owned, decentralised health delivery system with cross convergence at all stages to improve equal and fair delivery of maternal health care services such as antenatal care (ANC), skilled birth assistance (SBA), and post-natal care (PNC).The country has also witnessed the National Health Mission (NHM) programme in an extensive form of NRHM programme to evaluate the effectiveness of the NRHM in minimizing the gap of utilizing maternal and child health (MCH) services among various groups across the country. Even after such efforts made by the government, several studies have found that maternal health care utilisation has been disproportionally high among upper strata women where the poorest section has a little access over it [8–10]. Previous studies manifested that due to variations in the utilization of MCH services, huge disparities are observed in the occurrence of maternal deaths throughout the country [11, 12]. Studies suggested that education, wealth, and place of residence are the most significant factors which are affecting maternal health services [9, 13–15]. Apart from these, structural determinants, such as religion [16] and caste [17], are also significantly related to access and utilisation of maternal health services in India.

There are shreds of evidences which document the low utilization of MCH services among Muslims, compared to other religious communities in the country [18–20]. Muslims constitute the second largest religious community in the country, accounting 14.2 percent of the total population [16]. India has the third largest population of Muslims after Indonesia and Pakistan [16]. At the same time, Muslims are lagging in terms of human development indicators and mostly rely on the margins of basics of social, economic and political importance in India [20]. Additionally, Muslims exhibit more traditionalism and often experience exclusion in maternal care [21]. In fact, Muslim women often face stigma-related issues, and generally, do not participate in the society’s mainstream, which ultimately resulted in less participation in health services [22]. Many studies portray that education is meagre among Muslim women, and they are often far away from mass media exposure related to delivery and contraceptive uses [23, 24].

Further, fertility rate is very high among Muslims in India, and most of their population is living in low socio-economic position. Hence, low education and a lower standard of living often prevent Muslim women from accessing and utilising the maternal healthcare services. Several studies in India focused on the socio-economic determinants of maternal health care use among Muslim women [18–20]. Studies also focus on the inequality among subgroups in terms of utilization of various maternal health services due to the uneven distribution of wealth [14, 15]. But there is a lack of research focusing on the socio-economic inequality in the utilisation of maternal services among Muslims.

The main concern is how to improve maternal health services among the deprived, poor and disadvantaged sections of the society. In this context, it is essential to examine the rich-poor gap in maternal care utilisation and measuring the extent of inequality across the socio-economic groups among the Muslim population. With this outline, the present study attempts to accomplish two objectives. First, the study measures the disparities in maternal care utilisation between poor and non-poor Muslims. Second, this study explains the degree of contribution of some selected socio-economic factors to the inequality in utilisation of full antenatal care, skilled attendants at birth and postnatal care in India over time (2005-16).

## Methods

The study is based on cross-sectional survey data from the 3^rd^ (2005-06) and 4^th^ (2015-16) round of National Family Health Survey (NFHS). NFHS is a large-scale and multi-round survey that provides information about emerging Maternal and Child Health issues in India [25, 26]. The NFHS follows a multistage stratified random sampling design, and from each state, primary sampling units (PSU) are drawn by applying probability proportional to size (PPS) and systematic sampling methods.

The inequality has been measured for 14 states, namely Uttar Pradesh (UP), Bihar (BH), Maharashtra (MH), Andhra Pradesh (AP), Karnataka (KA), Uttarakhand (UK), Jharkhand (JH), Odisha (OR), Assam (AS), Gujarat (GJ), Himachal Pradesh (HP), Jammu & Kashmir (JK), West Bengal (WB) and Manipur (MN). The remaining states are excluded due to inadequacy in sample size.

### Defining variables

#### Outcome variables

The present study used three vital indicators of maternal health care: full antenatal care (ANC), skilled attendants at birth (SBA) and postnatal care (PNC) as outcome variables. The definition used in calculating each of the variables which are given below-

**Full antenatal care** is defined as women who had four or more visits for ANC, had at least one tetanus injection and consumed 100 IFA tablets/syrup for the last birth [20, 26].

**Skilled attendants at birth** is defined as the deliveries conducted either in a medical institution or at home assisted by a skilled person (doctor/nurse/Lady Health Visitor (LHV)/Auxiliary Nurse Midwife (ANM) for the last birth that occurred during the last five years preceding the survey [26, 27].

**Postnatal Care** includes those women who went for the check-up to any health facilities/doctors within 48 hours of delivery for their last birth. All indicators are based on the deliveries conducted in five years preceding the NFHS survey in both rounds [26].

#### Explanatory variables

The explanatory variables used in the study are wealth index, place of residence (urban, rural), age in years (15-24, 25-34 and 35-49), birth order (1, 2, 3 and above), education (no education, primary education, secondary education and higher education), caste (Scheduled Caste (SC), Scheduled Tribe (ST), Other backward Class (OBC), and Others) and mass media exposure (No, Yes).

### Statistical approach

This paper used the wealth index as a proxy measure of household economic status in the absence of income. NFHS provides five categories of wealth index, for instance, poorest, poorer, middle, richer and richest. Further, wealth index was categorizedas dichotomous variable i.e. poor (poorest and poorer) and non-poor (middle, richer and richest) to assess the wealth related differentialsin maternity care among Muslims.

For calculation of CI, wealth index has been divided into five equal quintiles, and for decomposition analysis, the continuous wealth index variable has been used. For decomposition analysis, a dummy of each variable has been generated, and place of residence (rural), age of women (15-24), birth order (first), women’s education (no education), caste (Others) and mass media exposure (No) have been taken as reference category.

#### Concentration index (CI)

The Concentration Index (CI) is applied to measure the health-related income inequality in full antenatal care, skilled attendants at birth and postnatal care. The concentration index is defined as the twice of the area between the concentration curve and the line of equality.

The CI curve plots the cumulative percentage of the health outcome variable (y-axis) on the cumulative percentage of the sample population, ranked by socio-economic status (x-axis). CI value varies from -1 to +1. A zero value of the concentration index indicates no inequality. A positive value of CI means pro-rich inequality and vice versa. When the CI curve lies below the line of equality, it demonstrates the disproportionate concentration of the outcome variables among the better-off and vice versa. The value of CI quantifies the extent of socio-economic inequality. The larger the absolute value, the more significant the inequalities.

1 $$\mathbf{C}=\frac{\mathbf{2}}{\boldsymbol{\mu}}\boldsymbol{\operatorname{cov}}\left({\boldsymbol{y}}_{\boldsymbol{i}},{\boldsymbol{R}}_{\boldsymbol{i}}\right)$$

Where ***C***is the concentration index; ***y***_***i***_ is the outcome variable index; ***R***_***i***_ is the fractional rank of individual, ***i*** in the distribution of socio-economic position; ***μ*** is the mean of the outcome variable of the sample, and ***cov*** denotes the covariance.

For the decomposition of CI, this paper used Wag-staff-type decomposition analysis [30], demonstrates that CI can be decomposed into the contributions of each factor to income-related health inequality. Each contribution is the outcome of the sensitivity of heath concerning that socio-economic factor and the extent of income-related inequality in that factor. Based on the linear regression, relationship between the outcome variable*y*_*i*_, the intercept*α*, the relative contribution of *x*_*ki*_ and the residual error ***ε***_***i***_in the equation 2,

2 $${\boldsymbol{y}}_{\boldsymbol{i}}=\boldsymbol{\alpha} +\sum {\boldsymbol{\beta}}_{\boldsymbol{k}}{\boldsymbol{x}}_{\boldsymbol{k}\boldsymbol{i}}+{\boldsymbol{\varepsilon}}_{\boldsymbol{i}}$$

Where *ε*_*i*_is an error term, given the relationship between *y*_*i*_ and *x*_*ki*_ in the equation 2, the CI for y (C) can be rewritten as in equation 3:

3 $$\boldsymbol{C}=\sum \left(\frac{{\boldsymbol{\beta}}_{\boldsymbol{k}}{\overline{\boldsymbol{x}}}_{\boldsymbol{k}}}{\upmu}\ \right){\boldsymbol{C}}_{\boldsymbol{k}}+\frac{\boldsymbol{GC}\boldsymbol{\upvarepsilon }}{\upmu}/\upmu$$

where*μ* is the mean of *y*_*i*_,${\overline{\ x}}_k$is the mean of *x*_*k*_, *β*_*k*_ is the coefficient from a linear regression of outcome variables, *C*_*k*_is the concentration index for *x*_*k*_ (defined analogously to*C*, and *GC*_ε_ is the generalized concentration index for the error term (*ε*_*i*_).

Equation (3) shows that C is the outcome of two components: first, determinants or ‘explained’ factors, equivalent to a weighted accumulation of the concentration indices of the regressors, where the weights are simply the elasticity, for instance, one unit change in the outcome variable is associated with one unit change in the explanatory variables. The explained factors indicate the proportion of inequalities in the outcome (full ANC, SBA, PNC) variable are explained by the selected explanatory factors, i.e.*x*_*k*_. Second, a residual or ‘unexplained’ factor $\Big(\frac{\boldsymbol{GC}\boldsymbol{\upvarepsilon }}{\boldsymbol{\mu}}/\boldsymbol{\mu}$**)** indicates the inequality in health variables that cannot be explained by selected explanatory factors across socioeconomic groups [28–30].

#### Marginal effect

Marginal effect refers to the one-unit change in dependent variable (outcome) is associated with one unit change in independent variable (explanatory factors) if all other independent factors are constant. It measures the change in outcome variable in respect to additional unit change in explanatory variable. For example, if the value of *x*_*i*_ change from *x*_*i*, 0_ to *x*_*i*, 1_ then the marginal value of the change in *x*_*i*_ will be following:

4 $$\varDelta {x}_i={x}_{i,1}-{x}_{i,0}$$

Then, marginal value of the change in *y*_*i*_ will be:

5 $$\varDelta y=f\left({x}_1,{x}_2,{x}_3,{x}_{i,1}\dots \dots \dots {x}_n\right)-f\left({x}_1,{x}_2,{x}_3,{x}_{i,0}\dots \dots \dots {x}_n\right)$$

Then,

6 $$\frac{\varDelta y}{\varDelta x}=\frac{\left({x}_1,{x}_2,{x}_3,{x}_{i,1}\dots \dots \dots {x}_n\right)-f\left({x}_1,{x}_2,{x}_3,{x}_{i,0}\dots \dots \dots {x}_n\right)}{x_{i,1}-{x}_{i,0}}$$

Where, $\frac{\varDelta y}{\varDelta x}$ is the marginal effect.

### Ethical considerations

The data for this study were taken from the secondary source called ‘MEASURE DHS’ archive (available from http://www.measuredhs.com). The researchers did not need to obtain ethical approval to use the data as the data were utterly anonymised and publicly available.

## Results

### Coverage of maternal health care services

The graph shows utilisation of maternal health care has doubled for each indicator during the last decade. However, the maximum increment was found in the utilisation of skilled attendants at birth. About three fourth of the Muslim women used skilled attendants at birth services in 2015-16 compared to 38.7 percent women in 2005-06. More than half of the women availed postnatal care in 2015-16 compared to one third women in 2005-06. The utilisation of full antenatal services has been increased from 9.3 percent in 2005-06 to 17.1 percent in 2015-16.

### Poor and non-poor disparities in maternal health care

The study revealed that poor and non-poor disparities in maternal care utilisation were comparatively higher in all socio-economic indicators (Table 1). Table 1 portrays that only 2.3 percent of poor mothers were using full ANC rather than 15 percent of non-poor mothers. Furthermore, 16.6 percent of poor are using SBA services compared with 57.9 percent of non-poor, while only 9.2 percent poor are using PNC services rather than 45.1 percent of non-poor Muslim.

**Table 1:** Poor and non-poor disparities in the utilisation of full antenatal care, skilled attendants at birth and post-natal care by selected socio-economic characteristics among Muslim women in India, 2005-06 and 2015-16

2005-06							2015-16					
	full ANC		SBA		PNC		full ANC		SBA		PNC	
Background variable	Poor	Non-Poor	Poor	Non-Poor	Poor	Non-Poor	Poor	Non-Poor	Poor	Non-Poor	Poor	Non-Poor
**Place of residence**												
Urban	4.3	15.8	23.3	70.3	14.6	56.5	11.2	28.0	64.6	88.2	43.8	69.7
Rural	2.1	14.3	15.9	44.8	8.6	33.1	6.2	20.4	58.3	81.8	36.8	65.1
**Age (in years)**												
15-24	2.9	15.9	21.5	60	13.2	46.8	9.3	25.4	66.1	87.0	41.8	68.0
25-34	2.4	16.1	14.1	58.4	7.0	46.1	6.5	25.6	58.5	85.6	38.1	68.4
35-49	1.1	6.8	12.0	45.9	6.0	34.6	3.3	20.8	46.7	80.7	28.4	65.2
**Birth order**												
1	3.4	23.7	30.7	74.4	18.6	58.3	11.1	28.0	72.3	90.2	47.8	73.9
2	4.5	17.7	19.8	61.4	12.6	52.3	7.9	28.2	60.4	87.3	40.1	69.2
3 and above	1.5	9.3	11.6	45.2	6.1	34.4	4.8	19.1	51.7	78.5	32.5	61.0
**Education**												
No education	1.5	4.5	14.4	34.2	7.9	20.9	4.3	9.8	52.9	71.1	33.0	54.8
Primary education	5.3	10.5	20.4	56.5	11.0	26.3	7.6	16.6	60.4	79.9	38.4	58.4
Secondary education	5.5	24.8	30.7	79.2	17.3	58.2	11.5	30.0	73.0	91.2	46.7	72.8
Higher education	0.0	39.3	0.0	94.9	0.0	88.4	30.0	39.5	86.1	95.9	68.0	79.2
**Caste**												
Schedule caste	0.0	1.8	12.9	35.0	5.3	22.3	1.7	18.8	56.3	84.3	30.8	57.8
Schedule tribe	2.9	13.4	19.9	36	11.3	33.5	7.1	20.3	61.1	84.4	37.3	63.3
OBC	1.7	15.5	15.4	57.1	7.2	44.1	5.0	26.3	58.4	83.7	38.3	67.0
Others	3.1	14.5	17.5	59.3	12.4	47.0	8.7	23.6	58.8	86.1	36.6	66.9
**Mass media exposure**												
No	1.2	3.3	14.9	31.0	8.5	20.1	5.2	10.3	53.4	69.6	32.4	52.1
Yes	3.9	17.9	19.1	65.4	10.3	51.5	9.4	27.0	68.0	88.0	45.3	70.0
Total	2.3	15.0	16.6	57.9	9.2	45.1	7.0	25.0	59.3	85.7	37.8	67.9

Table 1 also highlights a small volume of increment in maternity care utilisation among the poor Muslims, whereas non-poor mothers were utilising an enormous percentage of maternal care over the decade. The poor and non-poor gap marginallyreduced among rural women, whereas the gap remains high in urban areas. The non-poor rural Muslims (81.8 percent) were using more SBA services than poor Muslims (58.3 percent) and the PNC utilisation was 65.1 percent among non-poor and 36.8 percent among poor in rural women. Young poor mothers were using more maternal care than higher age group women. The findings also portray that women with three or more birth order were using higher percentage of maternity services.

The result shows that only 4.3 percent of poor women who had no education were availing full ANC compared to only 30 percent higher educated Muslims. More than half of (52.9 percent) poor uneducated women were using SBAs whereas it was 86.1 percent among poor high educated group. One third of poor mothers was using PNC as compared to 68 percent of higher educated mothers . In sum, education has influenced the rise in the utilization of all three critical indicators irrespective of economic status.

Moreover, Table 1 shows that non-poor women who had no mass media exposure, were using more maternal care service as compared to poor. However, non-poor Muslim who had any media exposure were using more maternity services, highlighting a wide gap in utilisation. Overall, table 1 portrays that only 7 percent of poor mothers are using full ANC rather than 25 percent of non-poor mothers. Almost 60 percent of poor are using SBA services as compared with 85.7 percent of non-poor, while only 37.8 percent poor were using PNC services rather than 67.9 percent of non-poor Muslims. Table 1 also highlights apoor and non-poor gap among the maternal care utilisation among Muslims in 2015-16.

### Inequalities in utilisation of maternal health care

**Fig. 1:**
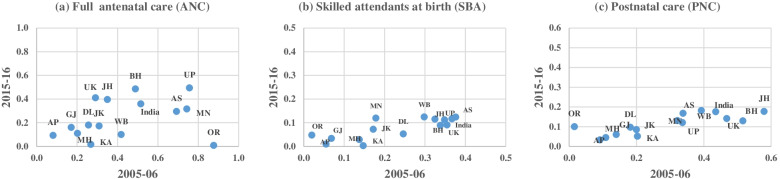
**a** Scatter plot of CI for full ANC (antenatal Care) for Muslim women in India, 2005-06 to 2015-16. **b**: Scatter plot of CI for SBA (skilled attendants at birth) for Muslim women in India, 2005-06 to 2015-16. **c**: Scatter plot of CI for PNC (postnatal care) for Muslim women in India, 2005-06 to 2015-16

Figure 1 (a, b and c) illustrates CI for the utilisation of full ANC, SBA and PNC among Muslim women in the selected states of India between 2005-16. The CI value for full ANC of India has declined from 0.515 in 2005-06 to 0.359 in 2015-16. Except in the Northern states, inequalities have been reduced in each state. The CI value has been reduced in Bihar (0.489 to 0.486), Assam (0.692 to 0.259), Jammu & Kashmir (0.309 to 0.173), Manipur (0.743 to 0.316), Uttar Pradesh (0.757 to 0.494), West Bengal (0.418 to 0.100) and Odisha (0.877 to 0.009) during 2005-06 to 2015-16. Odisha has shown astonishing reduction in inequality in full ANC in the last decade (Fig. 1 a). Inequality has been reduced sharply in the utilisation of SBA during the decade. CI value of SBA utilisation has declined to 0.116 in 2015-16 from 0.367 in 2005-06 for India. Although, inequality has been dropped below 0.1 in most of the states, but states like Bihar, Assam, Manipur, Uttar Pradesh, and West Bengal had substantial inequality in SBA utilisation (Fig. 1 b).

Figure 1 (c) portrays CI for the utilisation of PNC among Muslim women in the selected states of India between 2005-16. Similar to other two indicators of maternal care, inequality in postnatal care utilisation has also reduced during the period. However, the reduction in PNC was relatively less to SBA utilisation. Northern statescontributethe most inequality in PNC use. CI value of PNC utilisation declined to 0.176 in 2015-16 from 0.435 in 2005-06 for India.

**Fig. 2:**
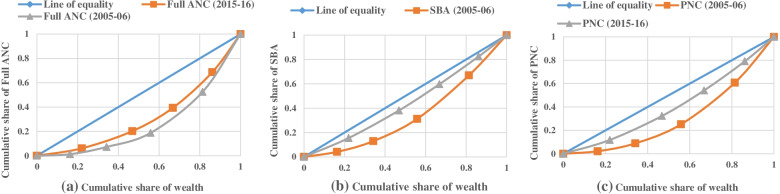
**a** Concentration curve showing ANC (antenatal Care) for Muslim women in India, 2005-06 and 2015-16. **b**: Concentration curve showing SBA (skilled attendants at birth) for Muslim women in India, 2005-06 and 2015-16. **c**: Concentration curve showing PNC (postnatal care) for Muslim women in India, 2005-06 and 2015-16

The Concentration curve for full ANC, SBA and PNC utilisation demonstrates graphical presentation of inequality depicted in Fig. 2(a), (b) and (c). Concentration curve shows that inequality has been reduced in each indicator of maternal care utilisation during 2005-16. However, inequality reduction in full ANC utilisation was comparatively less to SBA and PNC, which indicates much improvement in utilisation of maternal care in last decade among Muslims.

### Decomposition of inequalities (CI) in utilisation of maternal health care

Table 2 presents the estimates of marginal effect and decomposition analysis of inequality in the utilization of full ANC, SBA, and PNC among Muslim women in India, (2005-06). Secondary education made a decisive contribution of CI (0.079) to overall inequality, followed by higher education (0.022), mass media exposure (0.020), and three and more birth order (0.017) in full ANC. Similarly, in SBA utilisation, secondary education (0.166) followed by urban area (0.113), three and more birth order (0.053), mass media exposure (0.043) and higher education (0.036) contributed an absolute value to inequality. Furthermore, thesecondary education contributed a larger value of CI (0.170) to overall inequality followed by urban residence (0.092), higher education (0.045), three and more birth order (0.034) and media exposure (0.028) in PNC utilisation. The value of total explained inequality by these selected variables for full ANC was 0.132 and unexplained inequality (0.384) among Muslims. In addition, explained CI was 0.399 and residual was -0.033 for SBA utilisation. Correspondingly, the total explained inequality in PNC utilisation was 0.364 and unexplained inequality was 0.072 among Muslims in 2005-06.

**Table 2:** Estimates of marginal effect and decomposition analysis of inequality in the utilization of full ANC, SBA and PNC among Muslim women in India, (2005-06)

2005-06							
		Full ANC		SBA		PNC	
Explanatory variables	CI	Marginal Effect	Absolute Contribution to CI	Marginal Effect	Absolute Contribution to CI	Marginal Effect	Absolute Contribution to CI
Urban	0.427	-0.008*	-0.004	0.203***	0.113	0.166***	0.092
Age in years (25-34)	0.023	0.047***	0.002	0.079***	0.004	0.042*	0.002
Age in years (35-49)	-0.148	0.032*	-0.002	0.092***	-0.006	0.056*	-0.004
Birth order (2)	0.113	-0.031	-0.003	-0.108***	-0.011	-0.055*	-0.006
Birth order (3+)	-0.118	-0.069***	0.017	-0.210***	0.053	-0.136***	0.034
Primary education	0.025	0.066***	0.001	0.176***	0.003	0.138***	0.002
Secondary education	0.441	0.178***	0.079	0.373***	0.166	0.382***	0.170
Higher education	0.880	0.288***	0.022	0.476***	0.036	0.591***	0.045
Schedule caste	-0.105	0.049*	0.000	0.038	0.000	0.064	0.000
Schedule tribe	0.008	0.058***	0.001	0.063*	0.001	0.057*	0.001
OBC	0.002	0.042***	0.000	0.052	0.000	0.064*	0.000
Mass media exposure	0.210	0.039***	0.020	0.084***	0.043	0.055**	0.028
**Explained CI**			**0.132**		**0.399**		**0.364**
**Total CI**			**0.515**		**0.367**		**0.435**
**Residual**			**0.384**		**-0.033**		**0.072**

Table 3 shows that place of residence, birth order, education and mass media exposure were the prime contributor of inequality in maternal health care utilisation among Muslims in 2015-16 in India. However, the absolute value of contribution to inequality by these selected determinants has reduced in 2015-16 compared to 2005-06. Furthermore, in full ANC utilisation, secondary education (0.06) contributed in larger value of CI to inequality followed by three and more birth order (0.011), media exposure (0.031), higher education (0.037) and urban area (0.046). As far as SBA utilisation was concerned, it was mass media exposure (0.077) which contributed in most inequality followed by secondary education (0.069), urban area (0.045), higher education (0.031) and, three and more birth order (0.026) in SBA utilisation. Similarly, mass media exposure (0.083) contributed in greatest extent of CI to overall inequality followed by secondary education (0.63), urban area (0.043), higher education (0.032) and three and more birth order (0.03) in PNC use.

**Table 3:** Estimates of marginal effect and decomposition analysis of inequality in the utilization of full ANC, SBA and PNC among Muslim women in India, (2015-16)

	2015-16						
		Full ANC		SBA		PNC	
Explanatory Factors	CI	Marginal Effect	Absolute Contribution to CI	Marginal Effect	Absolute Contribution to CI	Marginal Effect	Absolute Contribution to CI
Urban	0.377	0.076***	0.046	0.075***	0.045	0.071***	0.043
Age in years (25-34)	0.034	0.019	0.001	0.024**	0.002	0.058***	0.004
Age in years (35-49)	-0.171	0.012	-0.001	-0.015	0.001	0.035*	-0.003
Birth order (2)	0.089	-0.004	0.000	-0.057***	-0.006	-0.06***	-0.006
Birth order (3+)	-0.161	-0.042**	0.011	-0.104***	0.026	-0.117***	0.03
Primary education	-0.104	0.037**	-0.003	0.062***	-0.004	0.034**	-0.002
Secondary education	0.251	0.148***	0.06	0.17***	0.069	0.156***	0.063
Higher education	0.683	0.258***	0.037	0.217***	0.031	0.225***	0.032
Schedule caste	-0.239	-0.026	0.001	0.006	0.000	-0.046	0.002
Schedule tribe	-0.195	-0.005	0.000	0.014	0.000	-0.009	0.000
OBC	0.039	0.021	0.002	0.011	0.001	0.035***	0.003
Mass media exposure	0.226	0.052***	0.031	0.13***	0.077	0.139***	0.083
**Explained CI**			**0.184**		**0.241**		**0.248**
**Total CI**			**0.359**		**0.116**		**0.176**
**Residual**			**0.175**		**-0.125**		**-0.073**

In spite of/despite reduction of inequality in maternity care among Muslims during the 2005-06 to 2015-16, a substantial extent of inequality remains in the same till date. It is equally important that the value of overall explained inequality in full ANC utilisation has been increased to 0.184, but a large extent of residual (0.175) remains also exist. Equally, explained CI was 0.241 and unexplained inequality was -0.125 for SBA utilisation. Additionally, the result also revealed that overall explained inequality in PNC utilisation was 0.248, and unexplained value of CI was -0.073 in India during 2015-16.

**Fig. 3:**
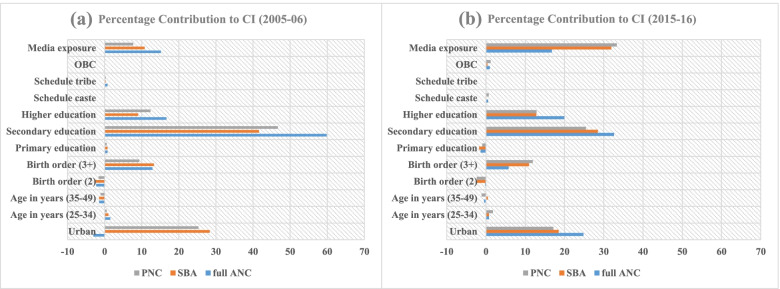
**a** Percentage contribution of CI for full ANC (antenatal Care), SBA (skilled attendants at birth) and PNC (postnatal care) among Muslim women in India, 2005-06. **b** Percentage contribution of CI for full ANC (antenatal Care), SBA (skilled attendants at birth) and PNC (postnatal care) among Muslim women in India, 2015-16

Figure 3(a) shows that secondary and higher education followed by urban area and media exposure contributed most percentage points to the inequality in maternal health care during 2005-06 among Muslim women. Figure 3(b) shows that percentage contribution of media exposure has been increased and highest in socio-economic inequalities in maternal care, followed by secondary education, urban residence and higher education during 2015-16 among Muslim Women.

## Discussion

This study focused primarily on the assessment of income-related health inequality in maternal care utilisation among Muslim women in India. Several studies have shown that India’s poor and socially marginalised population is still lagging behind and bearing the burden of health disadvantages [6–12]. According to census of India, Muslim account 14.2 percent share of the total population [16], yet constitute one of the most underprivileged and disadvantaged communities in India [17]. The NFHS data shows that the underutilization of maternal care is highest among Muslim women. Various studies have also showed that the rate of utilization of maternal healthcare is even poorer among the Muslim women, compared to the SC ST population. Even in a religious comparison, Muslims have the lowest access rate compared to Hindus, Jains, Christians and other religions.[7, 24]. Although there has been intervention in the past like NHM and initiatives taken by the government targeting the socially marginalised and poor people. The review of NHM reveals another picture with severe gaps in the availability, utilisation and affordability of maternal care [6]. The present study found that Muslim women living in urban areas are more likely to use maternal care compared to rural areas because of better accessibility of health facility. However, there was a large poor and non-poor gap in maternal care among urban residents rather than rural. Access to health care is very much asymmetric between rural and urban India. While urban residents choose between public or private providers, the rural residents had a few options [31, 32]. This is due to the health facilities provided by the public health spending which are often concentrated among the non-poor in urban areas [31, 32]. Although such facilities should be distributed equally across the groups, but the results show that poor rural Muslim women are utilizing more maternal health care compared to urban poor, which has been consistently increased in a decade. There have been various disputes about why Muslim utilization of maternal health care services is lower than that of other religious groups, and even lower than that of SC/ST communities. According to the discussion, this is related to the Muslim community's low socioeconomic condition and religious beliefs [33]. Because of their religious beliefs, many Muslim family members choose Muslim female doctors for routine healthcare check-ups during pregnancy and delivery [34, 35].

Maternal care utilization was significantly associated with the socio-demographic status of women. In the present study, factors such as birth order, education, caste, residence and mass media exposure appeared as the best predictor of the inequality in utilisation of maternal care. The findings have been consistent with the previous evidences [23, 32, 36, 37]. Birth order was strongly associated with the utilization of maternal care among Muslim women as they generally have higher fertility rate [36]. The results show that with the higher order of birth, utilisation of maternal care has been increasing among Muslim women. Although, in the first birth order, the poor and non-poor differential was considerably higher than the next birth order [38, 39] because women's age plays an important role [40, 41]. Mother's age may sometimes serve as a reflection for the women's build up knowledge of health care services, which may positively force on the use of health services. On the other hand, because of development in the healthcare delivery system and more educational opportunities for women in recent years, younger women might have an enhanced knowledge of available health care services and place more value upon modern medicine [20]. The gap between poor and non-poor in the utilization of maternity care was minimal among younger women, and poor-younger mothers in the Muslim community are utilising more maternal care services because the younger mother may be more educated and gives more care for first birth [42].

Further, the analysis also confirmed the significance of education, maternal care utilization is often associated with level of education of mothers, this is perhaps since educated women can access better health-related knowledge and awareness and tend to utilise it properly [37, 43]. In support, the results also show that irrespective of economic status, as the level of mothers' education increased, the utilization of MCH indicators also improved.

In India, social stratification is largely done on the basis of castes. Several studies suggest that women belonging to lower caste are often less interested to avail various maternal care as women belonging to lower castes negatively impact education, mass media exposure as well as the economic status of women [44–46]. Contrasting results were documented showing the poor Muslim women from the Scheduled Tribes are utilising more maternal care compared to SCs and OBCs. Furthermore, it was found that the poor and non-poor difference in utilisation of maternal care was most significant among OBC Muslims and comparatively less among SCs and STs [9, 13, 14]. Similarly, poor Muslims who had no mass media exposure were using more maternal care compared to non-poor. However, poor and non-poor disparities in maternity care utilisation was more among those who had mass media exposure [11, 12, 47, 48].

Further, to find out the main contributors to inequality in maternal care utilisation, the decomposition analysis was carried out and the findings from the analysis reveal that secondary education, mass media exposure, higher education and urban area are the main contributors. This study found an enormous unequal distribution of wealth, which was disproportionally concentrated particularly among urban residuals and higher educated women [9, 11–14, 31, 32, 35, 49]. It was also found that women with higher education, low mass media exposure [53,], high birth order, belonging to SCs and STs caste [50–52] have high marginal effects on the utilisation of maternal care with high wealth inequalities. Thus, the wealth inequality emerges to be the most dominant contributor of inequality in access to maternal care services [8, 14]. However, the other socio-demographic factors should also be considered in order to improve the overall maternal health care utilisation as the income inequalities are often operating on the inequalities in maternal care through these factors [5, 20].

## Conclusions

Most of the poor and non-poor differences in utilisation prevail among educated, urban resident, other backward caste. Moreover, utilisation of maternal health care has increased among poor Muslim mothers; the gap between poor and non-poor remains a more considerable extent across the Muslim community in the last decade. This study found most of the inequalities in maternal care utilisation are disproportionally concentrated among wealthier Muslims. Education and having mass media exposure are the most influential factors that contribute to the greatest extent of inequality in maternal care among Muslims in India.

Therefore, addressing more massive inequality contributed by education and wealth, the government should focus on education, especially among deprived caste and lower strata Muslims.

Access to mainstream media is the most significant driver to inequality, hence there is a need for mass media to be widely distributed among the Muslims. Another factor that contributes significantly to inequities in the utilization of maternal healthcare services is mother's education. There is a requirement to spread awareness about the value of women's education and imparting basic knowledge regarding the importance of maternal health care.Although having availability to schools, parents do not believe it is vital to educate their daughters.

However, while numerous initiatives and policies are in place to promote maternal healthcare services to achieve universal progress for the best interests of the marginalized groups, how well they are being implemented is a source of worry. Although it was believed that the primary techniques implemented under the National Rural Health Mission (NRHM) would improve the general quality of health care in India, it failed to achieve equality in service delivery throughout the country's states. There is a need to keep a closer eye on the ongoing programmes.

### Strengths and limitations of the study

The present study is not free from its limitations. The study did not consider the pregnancy outcomes in terms of non-live births i.e. abortion, miscarriage or stillbirth, which hinders us from talking about the maternity care utilisation with adverse outcomes in this group. The respondent’s report on maternity care utilisation, were entirely self-reported and under the recall-bias. The study failed to highlight some factors such as health care-seeking behaviour, health literacy, distant from the health care facility, provider bias and other system side factors due to data constraints in NFHS-4. We are also unable to study the women’s autonomy, measured by their control over financial, decision-making power and freedom of movement, and which may have a positive association with maternal health care utilization. Finally, the study only devoted to Muslim community and did not compare utilisation of services between Muslim households with other religious groups of women. Apart from these limitations, the present study is an attempt to present the status and determinants of maternity care utilization among Muslims using the data from the most recent large and nationally representative sample survey. This study is based on the more comprehensive critical reproductive indicators i.e. full ANC, SBAs and PNC, as against the only one indicator used in previous studies.

## Data Availability

The study is based on secondary data analysis. No data was collected for this study. The data are available for free on the DHS website (https://dhsprogram.com/what-we-do/survey/survey-display-355.cfm).

## Abbreviations

ANC: Antenatal Care
ANM: Auxiliary Nurse Midwives
CI: Concentration Index
IFA: Iron-Folic Acid
LHV: Lady Health Visitor
NHM: National Rural Health Mission
NRHM: National Rural Health Mission
OBC: Other backward Class
PCA: Principal Component Analysis
PNC: Postnatal Care
SBA: Skilled Birth Attendants
SC: Scheduled Caste
ST: Scheduled Tribe
WHO: World Health Organization
